# Investigation of Neuron Latency Modulated by Bilateral Inferior Collicular Interactions Using Whole-Cell Patch Clamp Recording in Brain Slices

**DOI:** 10.1155/2021/8030870

**Published:** 2021-12-10

**Authors:** Jinzhe Ma, Yangyang Han, Yiting Yao, Huimei Wang, Mengxia Chen, Ziying Fu, Qicai Chen, Jia Tang

**Affiliations:** School of Life Sciences and Hubei Key Lab of Genetic Regulation and Integrative Biology, Central China Normal University, Wuhan 430079, China

## Abstract

As the final level of the binaural integration center in the subcortical nucleus, the inferior colliculus (IC) plays an essential role in receiving binaural information input. Previous studies have focused on how interactions between the bilateral IC affect the firing rate of IC neurons. However, little is known concerning how the interactions within the bilateral IC affect neuron latency. In this study, we explored the synaptic mechanism of the effect of bilateral IC interactions on the latency of IC neurons. We used whole-cell patch clamp recordings to assess synaptic responses in isolated brain slices of Kunming mice. The results demonstrated that the excitation-inhibition projection was the main projection between the bilateral IC. Also, the bilateral IC interactions could change the reaction latency of most neurons to different degrees. The variation in latency was related to the type of synaptic input and the relative intensity of the excitation and inhibition. Furthermore, the latency variation also was caused by the duration change of the first subthreshold depolarization firing response of the neurons. The distribution characteristics of the different types of synaptic input also differed. Excitatory-inhibitory neurons were widely distributed in the IC dorsal and central nuclei, while excitatory neurons were relatively concentrated in these two nuclei. Inhibitory neurons did not exhibit any apparent distribution trend due to the small number of assessed neurons. These results provided an experimental reference to reveal the modulatory functions of bilateral IC projections.

## 1. Introduction

In the ascending auditory pathway, the central nucleus of the inferior colliculus (IC) receives and integrates excitatory and inhibitory inputs from many lower auditory nuclei. The IC also receives descending inputs from the auditory cortex through the cortico-collicular pathways [1]. Numerous projections also exist within the IC and bilaterally between the left and right IC, making the IC a critical center for information integration [2, 3]. In addition, no signal interactions take place between the bilateral medial geniculate bodies (MGB), establishing the IC as the final level in the binaural integration center in the subcortical nucleus, which is extremely important in the reception of binaural information [4, 5].

The anatomic study has established that the bilateral IC is connected by intercollicular commissural fibers (CoIC) [6–8]. The contralateral IC projections from the other side of the IC are primarily centered in the dorsal cortex of the inferior colliculus (DCIC) and the central nucleus of the inferior colliculus (CNIC), but few connections occur in the external nucleus of the inferior colliculus (ECIC) [9–12].

Currently, in vivo and in vitro electrophysiology studies have been used to investigate bilateral IC integration. In vitro studies have shown that focal electrical stimulation in CoIC induces both excitatory postsynaptic potentials (EPSPs) and inhibitory postsynaptic potentials (IPSPs). However, among all the PSP modes, the combination of IPSP/EPSP/IPSP is most common [13–15]. Pharmacological studies have proven that initial IPSPs result from single synapse inhibition, and subsequent IPSPs are likely due to coinhibition of multiple synapses [13]. Using focal drug injection [4, 16], electrical stimulation [17–20], and freezing one side of the IC [21–23], in vivo studies observed changes in response characteristics of the IC neurons located on the other side. These studies have proven that binaural IC interactions have considerable influence on the firing rate of IC neurons. However, little information concerning how latency is affected by bilateral IC interactions has been reported. Also, most of the previous studies about bilateral IC interaction have used extracellular recordings, which cannot elucidate synaptic mechanisms.

The central nervous system uses various means to encode sensory information. Besides firing rate coding, latency coding has attracted greater attention recently. Current studies have found that the response latency of neurons could modulate sensory characteristics [24–28]. Many basic acoustic parameters, like frequency, amplitude, and sound location, are thought to be encoded by first pulse latency, especially in the central auditory system. There have been reports that latency coding is faster, more precise, and could accommodate more information than firing rate coding [29]. In addition, latency coding and firing rate coding may handle different tasks. For example, latency coding might be used to identify what the stimulation is and then react swiftly. Meanwhile, the firing rate can convey the amount of stimulation that is occurring [27]. Therefore, each of these two coding modes cannot represent all of the information associated with acoustic pulse signals. Only a combination of both signals can successfully encode the acoustic pulse signal [30].

In addition, although most axons associated with the CoIC belong to IC neurons; the CoIC also contains many axons that emanate from the superior or inferior auditory center [7, 11, 31]. Thus, in vivo studies cannot completely exclude the impact from the subcortical pathway or corticofugal feedback circuit on bilateral IC integration.

In this study, we used whole-cell patch clamp recordings to explore synaptic mechanisms that might be involved in how bilateral IC interactions affect neural response latency. Thus, except for observations of synaptic input among neurons and variations in the subthreshold membrane potential, any impact from the subcortical pathway or corticofugal feedback circuit was eliminated, which enabled us to analyze the intercellular mechanism with more precision.

## 2. Materials and Methods

### 2.1. Animals and Brain Slice Preparation

Male and female Kunming mice (Mus musculus, Km; Hubei Research Center of Laboratory Animals, Wuhan, China) at postnatal days (P) 21 to 28 were used for this study. IC commissural brain slices were prepared according to the method described by Lee et al. [32]. To prepare the acute slices that contained the IC and the commissural connections, the mice first were deeply anesthetized using pentobarbital sodium. Then, the mice were decapitated, the skull was opened, the brains were quickly removed, and the brains were submerged in ice-cold, oxygenated, artificial cerebral spinal fluid (ACSF), which consisted of (in mm) 1.3 MgSO_4_, 5 KCl, 1.2 KH2PO_4_, 10 D-glucose, 26 NaHCO_3_, 120 NaCl, and 2.4 CaCl_2_. After the brain was removed, perform parasagittal blocking cut lateral to the IC, ~3.5 mm away from the medial longitudinal fissure. Following the first blocking cut, the brain was then rested along the parasagittal-blocked surface. Then a rostral cut was made at a 30° dorsoventral angle for preserving the commissural connections between the IC. Finally, the rostral cut surface was affixed to a vibratome cutting stage using instant adhesive glue (502) and sectioned at a thickness of 500 *μ*m in an ice-cold ACSF bath. Slices containing intact connections between the inferior colliculi were transferred to a holding chamber filled with oxygenated ACSF and incubated at 32°C for 1 h.

### 2.2. Electrophysiological Recordings

The IC slices were submerged in the recording chamber (RC-27, Warner, USA) using continuous bath perfusion of ACSF at 32°C. A homemade nylon net was used to fix the IC slices in place and prevent them from floating in the recording chamber. Oxygenated ASCF was continuously perfused (2–3 ml/min) into the recording chamber using a peristaltic pump (LEAD-2, Longer, China). Before whole-cell recording was initiated, the subnuclei including the CoIC in the IC were visually targeted for recording using an infrared microscope (BX-51, Olympus, Japan) with 5x lenses. The subnuclei locations were identified based on a mouse brain atlas. A stimulating concentric electrode (NEX-100, outer diameter of 0.25 mm, tip resistance 3–6 M*Ω*, Rhodes Medical Instruments, Woodland Hills, CA, USA) was used. The electrical pulse was performed using a patch clamp amplifier (EPC-10, HEKA, Germany) and modulated using a stimulus isolator (ISO-Flex, AMPI, Israel). Well-modulated electrical stimulation was transferred to the targeted nuclei via the concentric electrode.

**Figure 1:**
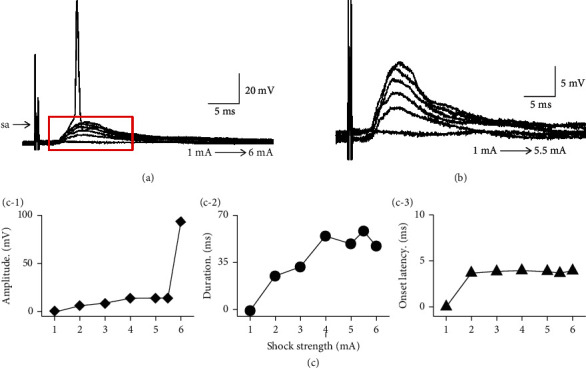
Relationship between the electrical stimulation strength and PSP variation in a typical IC neuron. (a) Synaptic response induced by a typical IC neuron (#20160330001-E-5-1) under different stimulation strengths. (b) Enlarged view of the area seen in the red box in (a). (c-1) Amplitude relative to stimulation signal strength. (c-2) Duration relative to stimulation signal strength. (c-3) Onset of latency relative to stimulation signal strength.

Two types of electrical stimulations were used, including single stimulations and train stimulations. The duration of each single stimulation was 0.5 ms, and the duration of each pulse in the train stimulations was 0.1 ms with a 0.9 ms interval, and 10 pulses were included in each train. The stimulus strength for these two types ranged from 0 to 6 mA [33]. To induce a synaptic response, the stimulation intensity and the PSP changes for each recorded neuron underwent amplitude response curve analysis (see Figure 1). Thus, the stimulation strength could be controlled in a moderate range that was less than the threshold for a synaptic response [15]. After the stimulating electrode was determined to be well placed, the microscope was focused on the contralateral IC. When the contralateral DCIC and CNIC were in focus, the microscope was switched to the 40x lenses and smooth, plump, pyramidal neurons with prominent axon were identified and clamped. When a gigaohm seal was achieved with the patch clamp system, negative pressure was used to break the cell membrane and perform whole-cell recording.

The recording electrode was constructed from borosilicate glass (inner diameter of 0.84 mm, outer diameter of 1.50 mm, WPI, USA). Before the electrophysiological recording was initiated, the recording electrode was filled with an intracellular solution containing (in mM) 108 K-gluconate, 8 Na-gluconate, 8 KCl, 2 MgCl_2_, 1 EGTA, 10 HEPES, 4 K-ATP, and 0.35 Na-GTP. After the electrode was filled, the tip resistance was 5 to 10 M*Ω*. The liquid junction potential was +10 mV and was compensated. The fast capacitance was compensated after the gigaohm seal was made, and the slow capacitance and series resistors were compensated after the membrane was broken. Series resistors were compensated when over 70% to avoid electromagnetic oscillation. The signal was amplified using a patch clamp amplifier (HEKA, Germany) and recorded using the signal acquisition system, PatchMaster (HEKA). The filter frequency was set to 10 kHz, and the sampling frequency was set to 20 kHz. The PatchMaster was used to instruct the patch clamp amplifier EPC-10 to modulate the intercellular potential or current stimulation and the membrane potential of the clamped neuron.

After these preparations were completed, the data were recorded using the following steps. First, after the whole-cell recording was performed, the CNIC was stimulated using the stimulation electrode and the PSCs and PSPs from the clamped neurons in the contralateral DCIC or CNIC were recorded under voltage-clamp or current-clamp modes, respectively. When recording under the voltage-clamp mode, the membrane potential was held at −60 mV and 0 mV to distinguish IPSPs and EPSPs , respectively [32, 34, 35]. Second, the neuron was current clamped with a depolarized step current (0–150 pA, in 10 pA steps, duration 100 ms) and a stimulation train was provided to the contralateral CNIC in the meantime. Then, we observed how the stimulation affected the excitatory responses induced by the input current. Third, after completing the electrophysiological recording, the stimulating electrode and recording electrode positions were recorded based on the locations of the micromanipulators.

### 2.3. Data Analysis

In this study, only neurons whose resting membrane potential was below −47 mV and the action potential was greater than 30 mV were used for analysis [15]. We defined a synaptic response as having a signal amplitude of three SDs or more over baseline [36]. Thus, the amplitude of the synaptic input was the deviation between three SDs from the baseline and the peak potential. The duration of the synaptic input was the time deviation between the two signals for which the amplitude was greater than three SDs from the baseline. When analyzing the integration of the bilateral IC, we focused on the variation of the first pulse latency. The first pulse latency was defined as the amount of time from when the depolarized current was given to the time of the peak of the first action potential. Then, the mean latency of eight repeated stimulations was analyzed. The latency variation was defined as the latency in the contrast that was 20% or greater under MT+30 stimulation. The duration of the subthreshold depolarization was defined as the time from when the depolarized current was given to the time that the threshold for the first action potential was attained.

The data were analyzed using Igor, Fitmaster, and Clamp, version 10.3; Spass, version 17.0, was used to analyze the variation in the data. SigmaPlot, version 10.0, was used to construct the graphs. The results were presented as means ± SD. Differences between groups were analyzed using paired *t*-tests.

## 3. Results

### 3.1. Synaptic Response Types in the Bilateral IC

**Figure 2:**
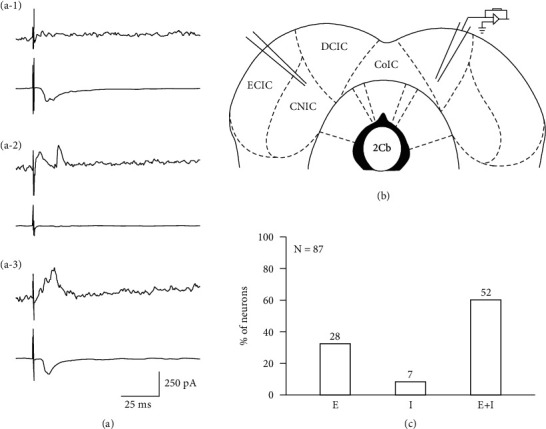
Synaptic input types between the bilateral inferior colliculus. (a-1–a-3) Showed a representative excitatory postsynaptic current (EPSC) (type E, #20160513001-E-5-1), inhibitory postsynaptic current (IPSC) (type I, #20160724001-E-5-1), and a combination of EPSC and IPSC (EPSC + IPSC) (type E + I, #20160801001-E-5-2), respectively, observed in the recording side of the CNIC and DCIC, which were recorded under −60 mV and 0 mV, respectively, using the voltage-clamp mode. (b) Pattern diagram illustrating bilateral IC stimulation and recording. (c) The proportions of these three types of synaptic inputs were 32.2% (28/87), 8% (7/87), and 59.8% (52/87).

**Figure 3:**
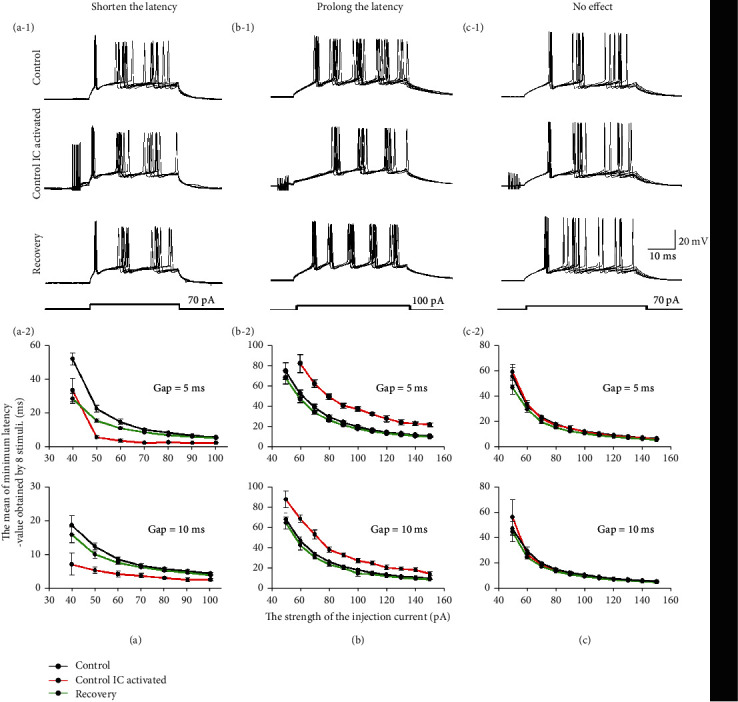
Influence of bilateral IC integration on latency. (a-1) The response of a typical neuron for which the latency was shortened (#20160513001-E-5-1) after stimulating the CNIC. The injected depolarizing current was 70 pA. (b-1) The response of a typical neuron for which the latency was prolonged (#20160702003-E-5-1) after stimulating the CNIC. The injected depolarizing current was 100 pA. (c-1) The response of a typical neuron for which the latency was unchanged (#20160801001-E-5-1) after stimulating the CNIC. The injected depolarizing current was 70 pA. (a-2, b-2, and c-2) The strength-latency function for three typical neurons for which the gap = 5 ms or the gap = 10 ms. The gap was defined as the time from when the stimulation in one side ended to the time the depolarizing current was injected. The black line indicates the strength-latency function for the control group. The red line indicates the strength-latency function when the synaptic input was induced from the contralateral IC neuron. The green line indicates the strength-latency function in recovery.

By stimulating one side of the CNIC, 87 neurons that exhibited synaptic connections were recorded on the other side of the DCIC and CNIC. When distinguishing excitatory potentials and inhibitory potentials by holding the membrane potential at −60 mV and 0 mV, respectively, and using the voltage-clamp mode, three types of synaptic inputs were recorded. These three inputs included excitatory postsynaptic currents (EPSCs, type E, Figure 2(a-1)), inhibitory postsynaptic currents (IPSCs, type I, Figure 2(a-2)), and a combination of EPSCs and IPSCs (EPSCs + IPSCs, type E + I, Figure 2(a-3)). The proportions of these three types were 32.2% (28/87), 8% (7/87), and 59.8% (52/87), respectively (Figure 2(c)).

### 3.2. Impact of Bilateral IC Integration on Latency

#### 3.2.1. The Recorded Neuron Latency Was Influenced by Inducing the Contralateral Synaptic Input

Depolarized step currents were provided to IC neurons on one side to induce neural excitation and stimulate the contralateral CNIC to explore how excitation or inhibition between IC neurons affected the response latency of the IC. Fifty-three neurons, including 18 type E, 4 type I, and 31 type E + I, neurons were recorded. After transferring to the recording side of the IC, the synapse inputs from the contralateral IC influenced the response latency of the recorded neurons to different degrees. The types of effects included shortening the latency (30.2%, 16/53), prolonging the latency (45.3%, 24/53), and no change in latency (24.5%, 13/53). Figures 3(a-1)–3(c-1) show examples of three typical neurons for which the latency was shortened, prolonged, and unchanged, respectively. If we take a neuron for which the latency was shortened as an example, after synaptic input from the contralateral IC was induced, the latency was significantly shortened compared to the control neurons. However, 10 min after the stimulation in the contralateral IC was removed, the neuron returned to the original latency.  Figure 3(a-1) shows the variation in latency after 70 pA depolarizing currents were injected in this neuron. In this study, we also recorded the variation in latency with different current strengths, which also is called the strength-latency function. The result suggested that the strength-latency function also shifted downward and would recover 10 min later if the stimulation in the contralateral IC was removed. This phenomenon was observed in neurons when the latency was prolonged or unchanged, similar to the observations for neurons in which the latency was shortened. Induction of synaptic inputs from one side of the IC prolonged or maintained the latency of recorded IC neurons and shifted up or maintained the strength-latency function (Figures 3(b-1), 3(b-2), 3(c-1), and 3(c-2)).

**Figure 4:**
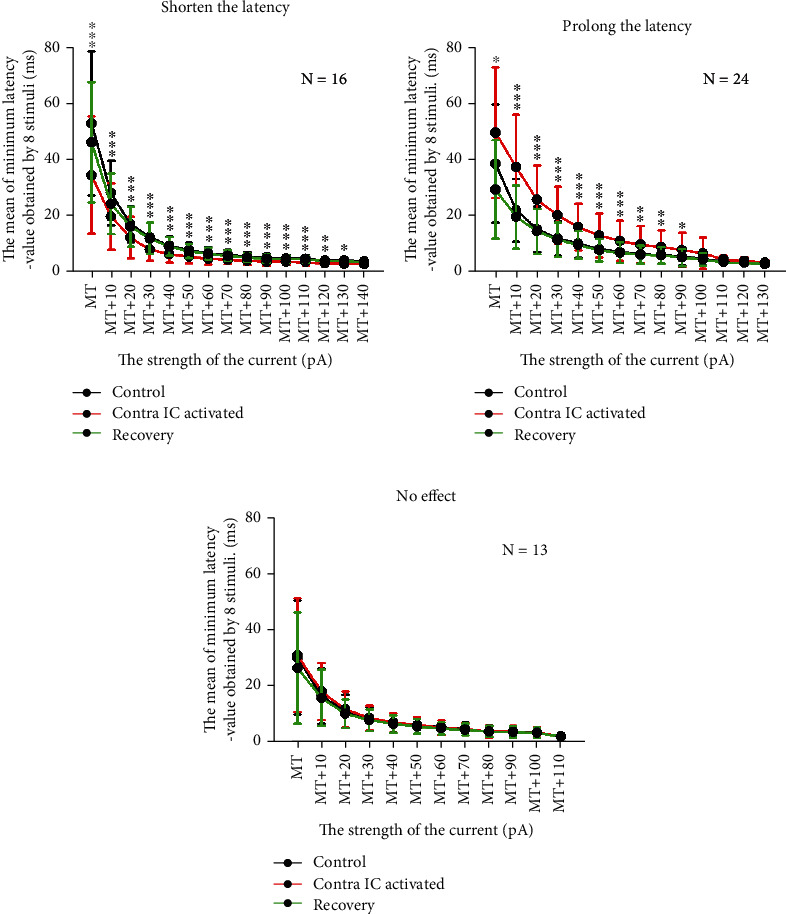
Variation in response latency in recording neurons under current strength step after induction of synaptic input from the contralateral IC. (a) Variation in the mean latency in 16 neurons exhibiting shortened latency under current strength that was above threshold. *N* indicates the number of neurons. (b) The variation in the mean latency in 24 neurons that exhibited prolonged latency under current strength that was above threshold. *N* indicates the number of neurons. (c) The variation in the mean latency in 13 neurons that did not exhibit any change in latency under current strength that was above threshold. *N* indicates the number of neurons. In (a–c), the black line represents the mean latency of the control group under current strength that was above threshold. The red line represents the mean latency under current strength that was above threshold when the synaptic input from the contralateral IC neuron was induced. The green line indicates the mean latency under current strength that was above threshold in recovery. The response latency after or before stimulation was analyzed statistically. ^∗∗∗^*P* < 0.001; ^∗∗^*P* < 0.01; ^∗^*P* < 0.05 (paired *t*-tests).

Figure 4 shows the statistical analysis of the variation in response latency under different current strength. We found that after electrical stimulation in lateral CNIC, response latency varied significantly under the most current strength for neurons for which the latency was prolonged or shortened (*P* < 0.001, paired *t*-test). In addition, most of neurons could recover to the control level 10 min later if the stimulation in the contralateral IC was removed. The results showed that the recovery time of each neuron was different after cessation of the contralateral stimulation. For neurons in which the latency was shortened, 75.0% (12/16) of neurons could recover within 10 min, while 25.0% (4/16) of neurons could recover after more than 10 min. For neurons in which the latency was prolonged, 87.5% (21/24) of neurons could recover within 10 min, while 12.5% (3/24) of neurons could recover after more than 10 min.

#### 3.2.2. The Relationship between the Type of Synaptic Input from the Bilateral IC and Latency Variation in the Contralateral Recording

In the preceding sections, we discovered that there were three types of synaptic inputs. When these synaptic inputs reach the recording side of the IC, the responses of the recorded neurons were changed to different degrees. Therefore, it remains to be determined whether there is any correlation between the types of synaptic input and the variation in response latency. The analysis demonstrated that the response latency variation induced by CNIC stimulation was indeed related to the type of synaptic input (Table 1). As seen in Table 1, most type E neurons exhibited shortened latencies (72.2%, 13/18). A few neurons exhibited unchanged latencies (22%, 4/18). Only one type of E neuron exhibited a prolonged latency (5.6%, 1/18). Half of the type I neurons exhibited prolonged latencies (50%, 2/4), and the remainder exhibited unchanged latencies (50%, 2/4). Most of the type E + I neurons exhibited prolonged latencies (67.7%, 21/31), while a few neurons exhibited shortened latencies (9.7%, 3/31) or unchanged latencies (22.6%, 7/31).

#### 3.2.3. The Characteristics of Type E + I Synaptic Input

**Figure 5:**
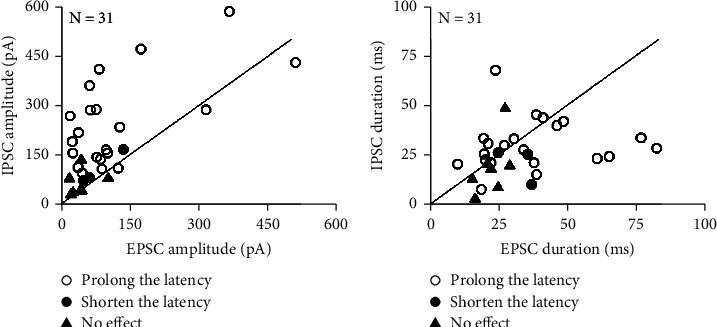
Comparison of the amplitude and duration of EPSCs and IPSCs from type E + I synaptic inputs. (a) The comparison of the amplitude of EPSCs and IPSCs from type E + I synaptic inputs. (b) The comparison of the duration of EPSCs and IPSCs from type E + I synaptic inputs. Empty circles indicate that the latency is prolonged. Solid circles indicate that the latency is shortened. Solid triangles indicate that the latency is unchanged.

It was noted that type E synaptic inputs shortened the latency through excitatory input. However, why do type E + I synaptic inputs, which include both excitatory and inhibitory inputs, still shorten, prolong, or have no effect on latency? To answer this question, we analyzed the response amplitude and duration of type E + I synaptic inputs induced by stimulating the contralateral CNIC.  Figure 5 shows the comparison of EPSCs and IPSCs from type E + I synaptic inputs with respect to amplitude (Figure 5(a)) and duration (Figure 5(b)). For most neurons in which the latency was prolonged due to induction by type E + I synaptic inputs, the IPSC and EPSC amplitudes were well above 150 pA (see the empty circles in Figure 5(a)), while the amplitudes of IPSC were far beyond EPSC. Only a few neurons were observed for which the E + I synaptic input shortened or had no effect on latency. For these neurons, the IPSCs were below 150 pA and equal or greater than the amplitudes of the EPSC (Figure 5, solid circles and solid triangles). After comparing the duration of EPSCs and IPSCs for neurons with type E + I synaptic inputs, we found that the duration of the IPSCs for most neurons was shorter than the EPSC duration. The duration of the IPSCs in a few neurons was equal to or longer than the duration of the EPSCs (Figure 5(b)).

#### 3.2.4. The Variations in Latency Caused by Variations in the Duration of Subthreshold Depolarization

**Figure 6:**
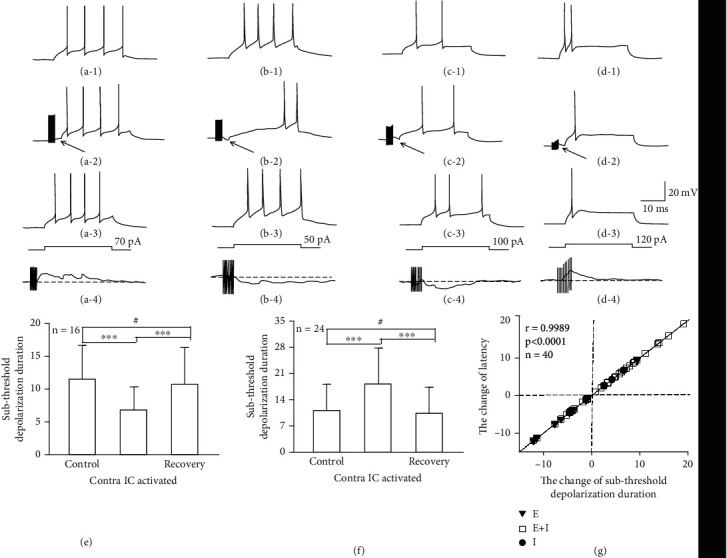
The influence of contralateral IC activation on subthreshold depolarization duration on the recording side. (a) A typical neuron exhibiting a shortened latency (#201607411002-E-5-1). (b–d) Three typical neurons exhibiting prolonged latencies (#20160613003-E-5-1, #20160903003-E-5-1, and #20160616002-E-5-1). 1–3 depict the subthreshold depolarization duration in control, contralateral activated, and recovery conditions, respectively. 4 is the neural response when the injected depolarized current is 0 pA, and synaptic input is induced in the contralateral IC. (e, f) Show the comparison of latency-shortened neurons and latency-prolonged neurons under MT+30 in control, contralateral IC activated, and recovery conditions. ^∗∗∗^*P* < 0.001; ^∗∗^*P* < 0.01; ^∗^*P* < 0.05 (paired *t*-tests). (g) Shows the correlation analysis of variation in latency and duration of the subthreshold depolarization. *r* is the correlation coefficient, and the solid line indicates the regression curve. *P* is the significance level.

After additional investigation into the latency variations of IC neurons, we observed that for some IC neurons under the current-clamp mode, stimulating the contralateral CNIC induced new EPSPs or IPSPs before firing. Therefore, were the PSPs the cause of the latency variation? We observed that EPSPs appeared in all neurons with shortened latencies before neural firing in the recording side of the IC (Figure 6(a-2); the EPSP is marked with an arrow, Figure 6(a-4), *n* = 16). Based on the type of synaptic response that occurred before firing, neurons with prolonged latency could be divided into three types. First, the IPSP appeared while the depolarizing current was injected or appeared after the depolarizing current was injected (Figure 6(b-2); the IPSP is marked with an arrow, Figure 6(b-4), 4/24). Second, both EPSPs and IPSPs appeared before neural firing but the IPSPs attained the recording side of the IC after the depolarizing current was injected. Therefore, only the EPSPs were observed before neural firing (Figure 6(c-2); EPSP is marked with an arrow, Figure 6(c-4), 9/24). Third, only EPSPs appeared before neural firing in IC neurons (Figure 6(d-2); EPSP is marked with an arrow, Figure 6(d-4), 11/24).

We observed that the appearance of the PSP shortened or prolonged the subthreshold depolarization duration of the first firing. Therefore, we performed additional statistical analysis on the subthreshold depolarization duration for IC neurons recorded under MT+30 in control, contralateral IC activated, and recovery conditions. We observed that the subthreshold depolarization duration for neurons with shortened latency was significantly shortened and the subthreshold depolarization duration for neurons with prolonged latency was significantly prolonged (Figures 6(e) and 6(f), control vs. contralateral IC activated, *P* < 0.001; contralateral IC activated vs. recovery, *P* < 0.001, paired *t*-tests). In this study, we also used correlation analysis to assess the variation in the subthreshold depolarization duration and latency and found significant correlations (Figure 6(g), *r* = 0.9989, *P* < 0.0001).

### 3.3. Distribution of Stimulating Sites and Recording Sites in the Bilateral IC

**Figure 7:**
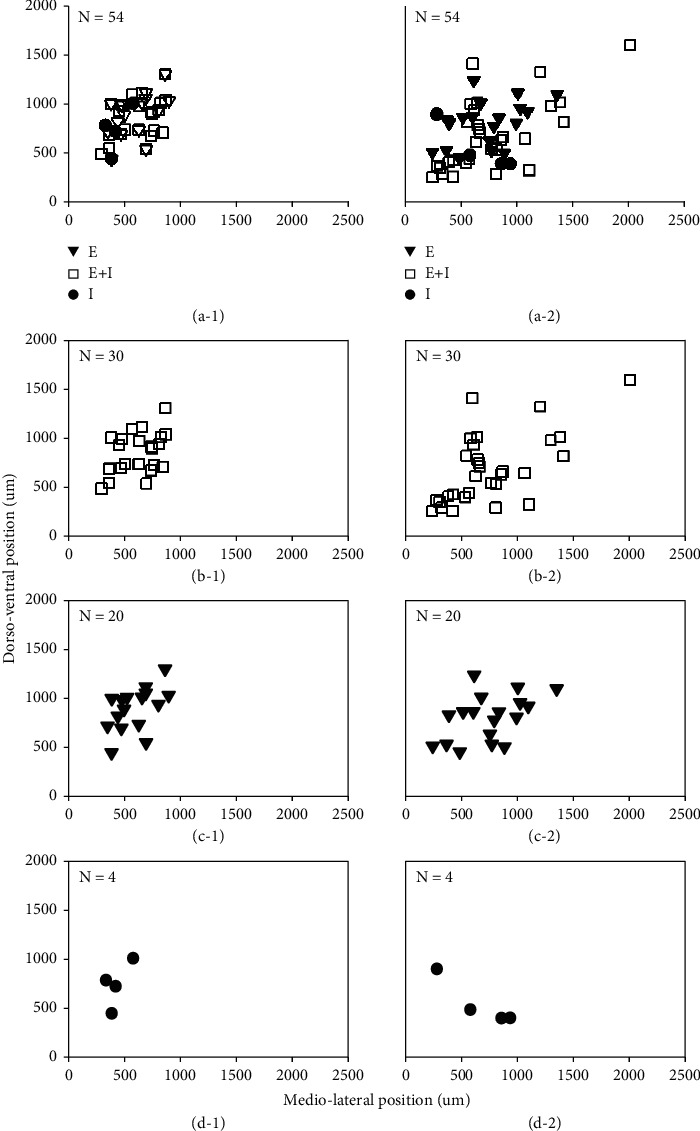
Distribution of stimulating and recording sites in the bilateral IC. The stimulating (a-1) and recording (a-2) sites for all neurons (*n* = 54). Stimulating (b-1) and recording (b-2) sites for type E + I neurons (*n* = 30). The stimulating (c-1) and recording (c-2) sites for type E neurons (*n* = 20). The stimulating (d-1) and recording (d-2) sites for type I neurons (*n* = 4). Rectangles indicate type E + I synaptic input. Triangles indicate type E synaptic input. Solid circles indicate type E synaptic input.

This study recorded the stimulating sites and recording sites of 54 IC neurons (see Figure 7). To observe their distribution characteristics efficiently, we followed the rule that centralized stimulations occurred on one side of the IC and extensively recorded on the other side. Therefore, the stimulating electrode needed to be placed close to the commissural fiber and extensive recording occurred in the CNIC and DCIC (Figures 7(a-1) and 7(a-2)). To observe the relationship between the types of synaptic input and the distribution of the stimulating and recording sites, we analyzed the three synaptic input types (Figures 7(b-1)–7(d-2)). We found that the recording sites for type E + I neurons were more scattered than the stimulating sites (Figures 7(b-1) and 7(b-2)). However, for type E neurons, the stimulating sites and recording sites were centralized (Figures 7(c-1) and 7(c-2)). Due to the low number of type I neurons that were recorded, the results did not reveal any significant regional localization (Figure 7(d-1)).

## 4. Discussion

In this study, we used whole-cell patch clamp recordings in isolated brain slices of Kunming mice to record synaptic responses to explore the synaptic mechanism underlying the effect of bilateral IC interactions on the latency of IC neurons.

### 4.1. Comparison of Synaptic Input Induced in the Contralateral CoIC and CNIC

The synaptic responses of the bilateral IC have been reported widely, but most studies have focused on stimulating the CoIC [13–15, 33]. Little research has focused on direct electrical stimulation of the contralateral CNIC. Some studies have reported that stimulation of the CoIC using the current-clamp mode could induce the following types of synaptic responses, single EPSP, single IPSP, EPSP followed by IPSP (EPSP/IPSP), complex responses (EPSP/IPSP/EPSP or IPSP/EPSP/IPSP), and others. Although the types of synaptic responses are complicated, there is evidence to suggest that after stimulating the CoIC, characteristic sequences such as IPSP/EPSP/IPSP are the primary synaptic response type [13]. In this study, we also recorded three types of synaptic responses, single EPSP, single IPSP, and complex types. However, the characteristic sequence for the observed complex type was not the same as seen with that for CoIC stimulation.

Another study reported that after electrical stimulation in the CoIC, the observed ratios of the three types of synaptic input sorted from largest to smallest were type E + I, type I, and type E when using the voltage-clamp mode [14]. However, in this study, when recording under the voltage-clamp mode and stimulating the contralateral CNIC, the ratio was different from the previous study. The synaptic inputs when sorted from largest to smallest, were type E + I (59.8%, 52/87), type E (32.2%, 28/87), and type I (8%, 7/87). The main difference was that the ratio of type I synapses was less than the ratio that resulted from electrical stimulation of the CoIC. This might be due to the different distributions of GABA-ergic neurons in the CoIC and CNIC. Another study found that the CoIC contains numerous GABA-ergic neurons, which are scarce in the CNIC [32]. This difference in GABA-ergic neurons might underlie the lack of direct inhibition and suggests that the function of the CoIC could enhance inhibition modulation of the bilateral IC.

### 4.2. Amplitude-Dependent Variation in Latency of IC Neurons

In this study, we observed that the latency of most neurons was inversely proportional to the strength of the injected depolarizing currents. Therefore, the higher the strength of the injection current, the shorter the response latency (Figures 3(a-2), 3(b-2), and 3(c-2)). This phenomenon was similar to “amplitude dependence in latency variation” observed in the in vivo study. In that study, when the sound amplitude increased, the latency of neurons was shortened significantly. Such variation has been demonstrated for visual [37–39], body [40], and auditory sensory [41–46] systems. Particularly in the auditory system, such “amplitude dependence in latency variation” has been observed in many nuclei of the auditory pathway, including the cochlear nucleus [45, 46], LSO [47], lemniscus lateralis [48], IC [48, 49], MGB [50], and AC [43]. In this study, we came to the same conclusion by injecting depolarizing currents of different strengths in clamped neurons in brain slices and obtained additional evidence of such latency variations.

### 4.3. Possible Synaptic Mechanism Underlying Bilateral IC Interactions Influencing Latency

What was the underlying reason that electrical stimulation in one side of the CNIC induces latency variations in 75.5% of the neurons on the other side of the IC (shortened latency, 30.2%, 16/53; prolonged latency, 45.3%, 24/53)? In this study, latency was composed of two parts. One part was the time deviation from the time the depolarizing current was injected to the time that first firing reached threshold, which is called the subthreshold depolarization duration. The other part was the time deviation from the time that the first firing reached the threshold to the time that the first firing reached the peak, which is called the rise time. The results from this study suggested that the variation in latency was caused by the variation in the subthreshold depolarization duration (Figure 6(c)). Thus, when focal electrical stimulation occurred in the contralateral CNIC, the induced variation in the subthreshold depolarization duration could be explained as the result of variation in the membrane potential. For neurons that exhibited a shortened latency, EPSPs induced by contralateral stimulation appeared before the firing response in IC neurons (Figure 6(a-2), marked with a red arrow, Figure 6(a-4), *n* = 16). The appearance of EPSPs shortened the deviation between the resting potential and the threshold potential, which decreased the subthreshold depolarization duration and shortened the response latency in the neuron. Neurons that exhibited prolonged latency could be divided into three types. First, only IPSPs appeared while injection of a depolarizing current or the IPSP appears after the injection of a depolarizing current (Figure 6(b-2), marked with a red arrow, Figure 6(b-4), *n* = 4/24). Second, both EPSPs and IPSPs appeared before firing in the recording side of the IC. However, the time that the IPSP reached the IC of the recording side was immediately after the injection of the depolarizing current. Thus, only EPSPs were observed before neural firing (Figure 6(c-2), marked with a red arrow, Figure 6(c-4), 9/24). Third, before firing in the IC neurons in the recording side, only EPSPs appeared (Figure 6(d-2), marked with a red arrow, Figure 6(d-4), 11/24). Although IPSPs in these neurons were not recorded in this study, IPSPs possibly could take place. Concerning the resting potential, the ion channel that modulates the IPSPs is closed, which made it challenging to distinguish IPSPs, or the IPSP might appear in the form of a depolarization [51]. In conclusion, for neurons with prolonged latency, the appearance of IPSPs strengthened the potential deviation between the resting potential and the threshold potential, which prolonged the duration of the subthreshold depolarization and then prolonged the latency of neuron.

There are two possible reasons why the remaining 24.5% of IC neurons stimulated in the contralateral CNIC did not exhibit changes in response latency. First, these neurons might not participate in latency coding but function in other aspects of auditory analysis. Second, the amplitude of the synaptic input might be too small to affect the excitatory response of neurons on the recording side (Figure 5(a)). We also observed a relationship between the types of synaptic input and variation in latency (Table 1). For most type E neurons, the synaptic input shortened the latency (72.2%, 13/18), and for most type E + I neurons, synaptic input prolonged the latency (67.7%, 21/31). For type I neurons, the latency exhibited by half of the neurons of the recording side was prolonged (50%, 2/4) and the other half was unchanged (50%, 2/4). The following model can explain these phenomena [52].

First, we consider the situation that most synaptic input from type E + I neurons on the recording side prolonged the latency of neurons on the recording side. Such neurons received three kinds of synaptic input. Excitatory input was produced by self-injected currents, and excitatory and inhibitory inputs were produced by focal electrical stimulation in the contralateral IC. We assumed that the three kinds of synaptic inputs reached the IC neurons on the recording side almost at the same time and the duration of excitatory input exceeded the inhibitory input (the duration of the injected depolarizing current was 100 ms, but for most neurons, the duration of the EPSC or IPSC in the type E + I synaptic input was less than 100 ms, and the duration of the IPSC was less than the EPSC (Figure 5(b)). Therefore, the excitatory and inhibitory inputs overlapped for a time. In this overlapping period, the amplitude of the inhibitory input for most neurons was larger than the amplitude of the excitatory input. The amplitude of the injected depolarizing current varied between 0 and 150 pA, but for most neurons, the amplitude of the IPSC in the type E + I synaptic input was much higher than 150 pA and higher than the amplitude of the EPSC (Figure 5(a), marked as empty circles). Therefore, the range of variation in the latency could be due to variation in the overlapping period of excitatory and inhibitory inputs. The overlapping period was different for each neuron. For example, the longer the inhibition, the longer the overlapping period. Inversely, the shorter the inhibition, the shorter the overlapping period. Such differences altered the range of variation in latency exhibited by the neurons. This model can explain the phenomena for which type I synaptic inputs prolong the response latency.

Concerning the phenomenon in which most type E synaptic inputs shorten the latency of neurons on the recording side, we predicted that this type only receives excitatory input, including excitatory synaptic-induced input from self-injected currents and focal electrical stimulation in the contralateral IC. The sum of these two excitatory inputs would shorten the response latency of excitatory modulated neurons in the recording side.

### 4.4. Centralized Excitatory and Extensive Inhibition in the Bilateral IC

After recording at stimulating and recording sites, we found that type E + I neurons were extensively distributed in the CNIC and DCIC (Figures 7(b-1) and 7(b-2)), type E neurons were centralized (Figures 7(c-1) and 7(c-2)), and type I neurons exhibited no localized distribution characteristics since the neuron number was small (Figures 7(d-1) and 7(d-2)). The lateral extracellular study in our lab found that bilateral IC interaction can function not only in the corresponding frequency layer but also in the frequency layer that does not correspond in the bilateral IC, which also shows centralized excitatory and extensive inhibition [17, 19]. Most type E synaptic inputs in this study shortened the latency of neurons in the recording side (72.2%, 13/18), and most type E + I synaptic inputs prolonged the latency of neurons in the recording side (67.7%, 21/31; Figure 2). Therefore, we considered this evidence to prove centralized excitatory and extensive inhibition in the bilateral IC.

### 4.5. Biological Significance of the Effect of Bilateral IC Interactions on the Latency of Neural Responses

Previous studies concerning how bilateral IC interactions affect the sound frequency and amplitude primarily have focused on neuron firing rates. Studies also found that after electrical stimulation on one side of the IC, the amplitude firing rate function and frequency response area of the other side enhanced the ability to process frequency and amplitude information [17, 19, 22]. Also, bilateral IC interactions can facilitate modulation by regulating the firing rate [4, 16]. The central neural system can encode sensory information in different forms. Except for firing rate coding, latency coding is present in sensory coding. Studies have reported that latency coding is faster and more precise and carries more information relative to sound pulse [29]. These two ways of coding are thought to handle different tasks. However, only the combination of firing rate coding and latency coding can encode sensory information well [27]. In this study, we observed that bilateral IC interactions could shift the amplitude-latency function up or down compared to the control group (Figures 3(a-2) and 3(b-2)), demonstrating that amplitude analysis can be well modulated by the response latency of IC neurons. Also, bilateral IC integration changed the neural response latency for 75.5% of the IC neurons, which indicated that bilateral IC interaction is a process that started before the action potentials. This process strengthened the response in the contralateral IC. Thus, this modulation in the bilateral IC is critical in binaural input.

## 5. Conclusions

According to this study, we concluded that the excitation-inhibition projection was the main projection between the bilateral IC. Also, the bilateral IC interactions could change the reaction latency of most IC neurons to different degrees. The variation in latency was related to the type of synaptic input and the relative intensity of the excitation and inhibition. Furthermore, excitatory-inhibitory neurons were widely distributed in the IC dorsal and central nuclei, while excitatory neurons were relatively concentrated in these two nuclei. Inhibitory neurons did not exhibit any apparent distribution trend due to the small number of assessed neurons. These results provided an experimental reference to reveal the modulatory functions of bilateral IC projections.

## Figures and Tables

**Table 1:** The relationship between the type of synaptic input from the bilateral IC and latency variation in the contralateral recording.

	Prolong the latency	Shorten the latency	No effect
E	1	13	4
I	2	0	2
E + I	21	3	7

## Data Availability

The data used to support the findings of this study are included within the article.
